# Bioactivity-Guided Fractionation of Physical Fatigue-Attenuating Components from *Rubus parvifolius* L.

**DOI:** 10.3390/molecules180911624

**Published:** 2013-09-23

**Authors:** Jianhong Chen, Xianfeng Wang, Yongqing Cai, Ming Tang, Qing Dai, Xiaogang Hu, Mingchun Huang, Fengjun Sun, Yao Liu, Peiyuan Xia

**Affiliations:** Department of Pharmacy, Southwest Hospital, Third Military Medical University, Chongqing 400038, China; E-Mails: chenjh-110@263.net (J.C.); wxf3111@126.com (X.W.); cy0721@163.com (Y.C.); tangmin1007@163.com (M.T.); dq050@163.com (Q.D.); hxgcq1987@126.com (X.H.); xyjk11@sina.com (M.H.); fengj_sun@163.com (F.S.); lylyl1982@hotmail.com (Y.L.)

**Keywords:** *Rubus parvifolius* L., anti-fatigue, macroporous resin, weight-loaded swimming

## Abstract

Alleviation of fatigue has been emerging as a serious issue that requires urgent attention. Health professionals and sports physiologists have been looking for active natural products and synthetic compounds to overcome fatigue in humans. This study was designed to define the anti-fatigue property of *Rubus parvifolius* L. (RPL) by characterization of active constituents using a mouse forced swimming test model. Four RPL fractions with different polarities containing anti-fatigue activity were sequentially isolated from the *n*-butanol RPL extract, followed by elution of 50% ethanol-water fraction from D101 macroporous resin chromatography to obtain nigaichigoside F_1_, suavissimoside R_1_ and coreanoside F_1_. Active constituents of the 50% ethanol-water eluate of RPL were total saponins. The fractions were examined based on the effect on weight-loaded swimming capacity of mice. Serum levels of urea nitrogen (SUN), triglyceride fatty acids (TG), lactate dehydrogenase (LDH), lactic acid (LA), ammonia and hepatic glycogen (HG) were also examined for potential mechanisms underlying the anti-fatigue effect of RPL extracts. During the experiment, two inflammatory markers, interleukin-6 (IL-6) and tumor necrosis factor (TNF-α) in serum, were measured. We found that total saponins from RPL possess potent capabilities to alleviate mouse fatigue induced by forced swimming and that nigaichigoside F_1_ was responsible for the pharmacological effect. The underlying mechanisms include delays of SUN and LA accumulation, a decrease in TG level by increasing fat consumption, increases in HG and LDH so that lactic acid accumulation and ammonia in the muscle were reduced, and suppression of increased immune activation and inflammatory cytokine production. Our findings will be helpful for functional identification of novel anti-fatigue components from natural medicinal herbs.

## 1. Introduction

*Rubus parvifolius* L. (RPL), belonging to the genus *Rubus*, is a deciduous thorny shrub, native to the eastern and southern regions of Asia. It has been used as an anti-inflammatory and anti-infectious herbal medicine for treatment of a variety of diseases including rheum, dysentery, enteritis, angina, hepatitis, rheumatism, dermatitis and eczema [1]. Previous studies demonstrated that RPL extracts exhibited antioxidant capacity [2,3,4], neuroprotective effects [5,6] and anti-tumor properties [7].

Fatigue is defined as difficulty in initiating or sustaining voluntary activities, resulting from severe stress and hard physical or mental work [8,9]. Therefore, alleviation of fatigue has become a serious issue that requires urgent attention. In the past few decades, health professionals and sports physiologists have been looking for active natural products and synthetic chemicals to postpone, and accelerate the elimination of, fatigue in humans [10]. However, effective drugs with few side effects are limited. The aim of the present study was to examine the anti-fatigue properties of the aqueous ethanol extract of RPL, its fractions and isolated compounds to provide a scientific basis for the industrial production and clinical use of RPL in a mouse forced swimming test model. We further examined the levels of mouse serum urea nitrogen (SUN), triglyceride fatty acids (TG), lactate dehydrogenase (LDH), lactic acid (LA), ammonia and hepatic glycogen (HG) to elucidate the mechanisms underlying the anti-fatigue effect of RPL. Two inflammatory markers interleukin-6 (IL-6) and tumor necrosis factor (TNF-α) in serum were also examined.

## 2. Results and Discussion

### 2.1. Chemical Analysis

**Figure 1:**
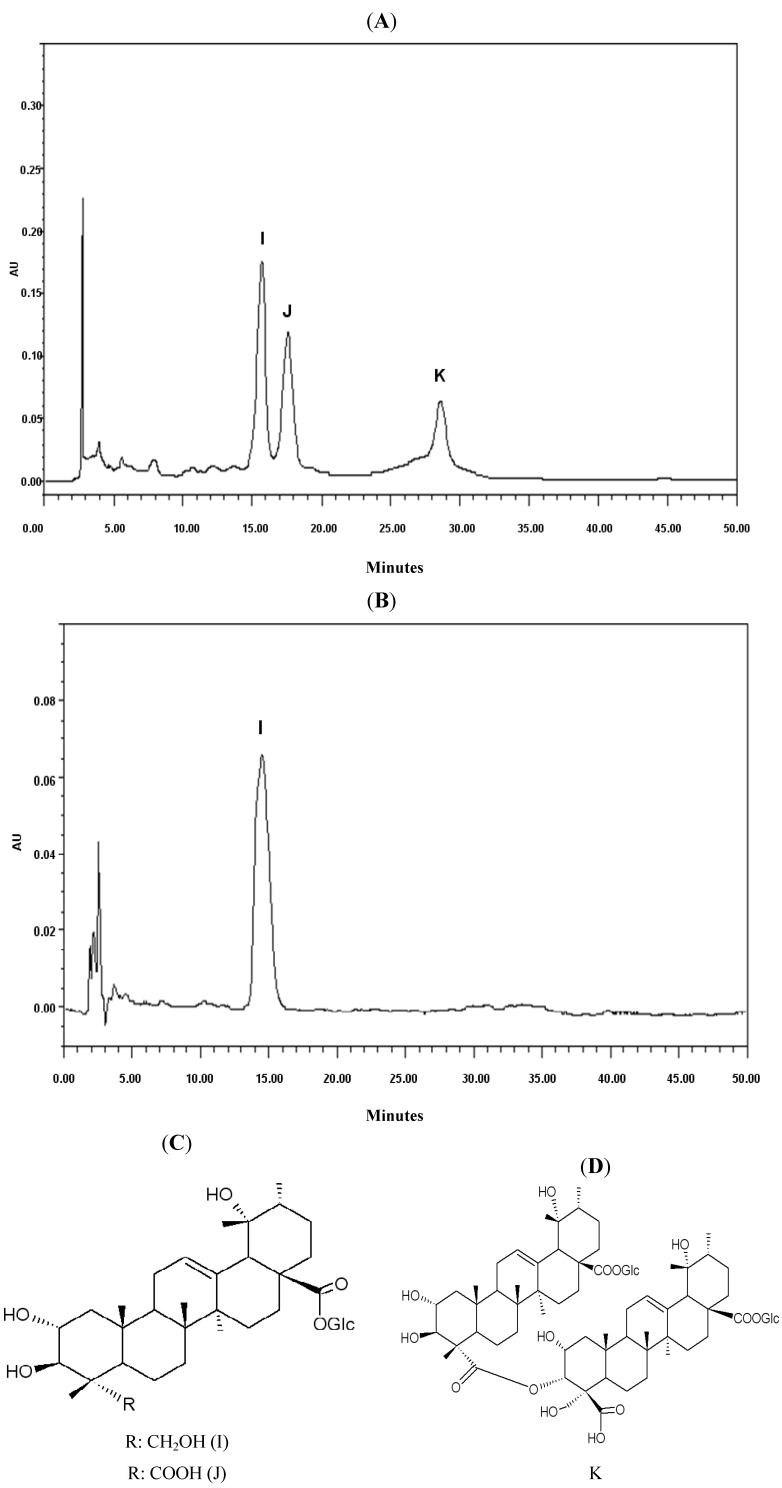
HPLC fractions from *Rubus parvifolius* L. (**A**) HPLC fingerprint of 50% ethanol fraction. (**B**) HPLC of 50% ethanol fraction isolate (*nigaichigoside F_1_*). (**C**) Chemical structure of *nigaichigoside F_1_* (I) *suavissimoside R_1_* (J). (**D**) Chemical structure of *coreanoside F1* (K)*.*

From the 50% ethanol fraction eluated from macroporous resin, three saponins (Figure 1A were obtained and identified as nigaichigoside F_1_ (compound I, Figure 1B,C), suavissimoside R_1_ (compound J, Figure 1C) and coreanoside F_1_ (compound K, Figure 1D), both of which have been previously reported and isolated from the leaves of *Rubus* species [11,12,13]. The structures were elucidated unambiguously by comparing experimental data with literature data.

### 2.2. The Effects of RPL on Mouse Body Weight Change

We compared the effects of red ginseng and different solvent fractions of RPL on mouse body weight at initial and terminal stages of the swimming test. As shown in Table 1, at the end of administration, there was an increase in the body weight of the mice in all groups. 

No significant difference in the body weight was found in treatment groups as compared with control group during initial and terminal stages of the animal experiments I and II. The effects of *n*-butanol fractions and nigaichigoside F_1_ on body weight were also compared.

**Table 1:** The effects of the fractions of RPL and red ginseng on mice body weights.

Groups	Body weights(g)		
	Initial	Final	Increased
Control	23.8 ± 0.5	40.2 ± 0.8	16.4 ± 0.8
Red Ginseng	24.0 ± 0.7	39.6 ± 0.8	15.6 ± 0.9
A1	24.4 ± 1.0	40.5 ± 0.9	16.1 ± 1.2
A2	23.7 ± 0.9	39.6 ± 1.3	15.9 ± 1.2
B1	24.2 ± 0.9	39.8 ± 1.3	15.6 ± 1.0
B2	23.6 ± 0.9	39.3 ± 1.1	15.7 ± 0.8
C1	24.2 ± 1.0	40.6 ± 1.2	16.3 ± 0.9
C2	23.3 ± 0.9	39.5 ± 1.2	16.1 ± 1.4
D1	24.5 ± 1.0	41.1 ± 1.3	16.6 ± 1.1
D2	24.1 ± 0.8	39.9 ± 1.2	15.8 ± 1.2

A1: low dose of petroleum ether fraction; A2: high dose of petroleum ether fraction; B1: low dose of ethyl acetate fraction; B2: high dose of ethyl acetate fraction; C1: low dose of *n*-butanol fraction; C2: high dose of *n*-butanol fraction; D1: low dose of water reside; D2: high dose of water reside; Low dose denotes 20 mg/kg; High dose denotes 40 mg/kg for each mouse.

As shown in Table 2, there were no significant differences in the body weight of the treatment groups as compared with the control group during initial and terminal stages in the mouse experiments I and II, although there were increases in the body weight of the mice in all groups. Thus, the compounds and derivates do not change the natural body weight gain of mice.

**Table 2:** The effects of the *n*-butanol fraction and nigaichigoside F_1_ on mice body weights.

Groups	Body weights(g)		
	Initial	Final	Increased
Normal	23.2 ± 0.6	31.2 ± 0.8	8.1 ± 0.7
Control	23.3 ± 0.5	31.3 ± 0.6	8.0 ± 0.6
RG1	23.8 ± 0.8	31.2 ± 0.7	7.4 ± 0.8
RG2	24.0 ± 0.7	31.9 ± 0.6	7.9 ± 0.8
E1	23.7 ± 0.9	31.6 ± 0.9	7.9 ± 0.7
E2	23.9 ± 0.9	31.6 ± 0.7	7.7 ± 1.0
F1	22.8 ± 0.8	31.2 ± 0.6	8.4 ± 1.1
F2	23.5 ± 0.7	30.9 ± 0.6	7.4 ± 0.7
G1	23.7 ± 0.9	31.0 ± 1.0	7.3 ± 0.8
G2	23.2 ± 0.8	30.9 ± 0.9	7.7 ± 0.9
H1	23.1 ± 0.8	30.6 ± 0.8	7.6 ± 0.7
H2	22.9 ± 0.8	30.8 ± 0.9	7.9 ± 1.1
I1	24.0 ± 1.0	32.1 ± 1.0	8.1 ± 0.9
I2	23.7 ± 0.8	31.1 ± 0.9	7.5 ± 0.9

Normal: non-swimming control group; Control: swimming control group; RG1: low dose of red ginseng; RG2: high dose of red ginseng; E1: low dose of water eluate; E2: high dose of water eluate; F1: low dose of 20% ethanol eluate; F2: high dose of 20% ethanol eluate; G1: low dose of 50% ethanol eluate; G2: high dose of 50% ethanol eluate; H1: low dose of 100% ethanol eluate; H2: high dose of 100% ethanol eluate; I1 and I2 mean that animals were treated with nigaichigoside F_1_ 2 mg/kg and 4 mg/kg.

### 2.3. The Effects of RPL on Mice in Swimming-to-Exhaustion Test

**Figure 2:**
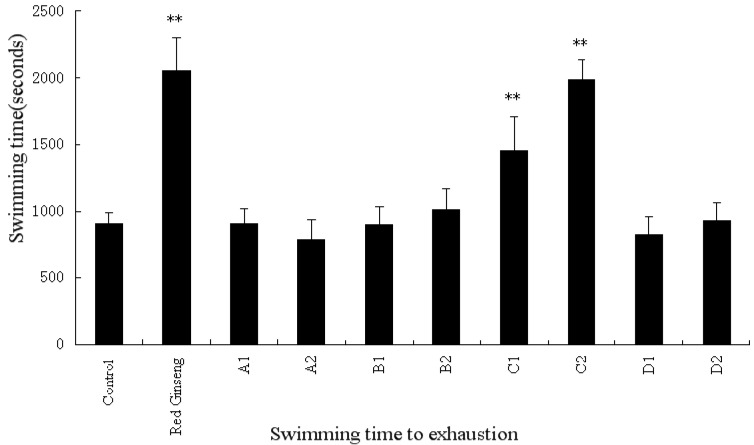
The effects of isolated fractions from *Rubus parvifolius* L. and red ginseng on swimming time to exhaustion of weight-loaded mice. A1: low dose of petroleum ether fraction, A2: high dose of petroleum ether fraction, B1: low dose of ethyl acetate fraction, B2: high dose of ethyl acetate fraction, C1: low dose of *n*-butanol fraction, C2: high dose of n-butanol fraction, D1: low dose of water reside, and D2: high dose of water reside. Low dose denotes 20 mg/kg per mouse, and high dose denotes 40 mg/kg per mouse.

**Figure 3:**
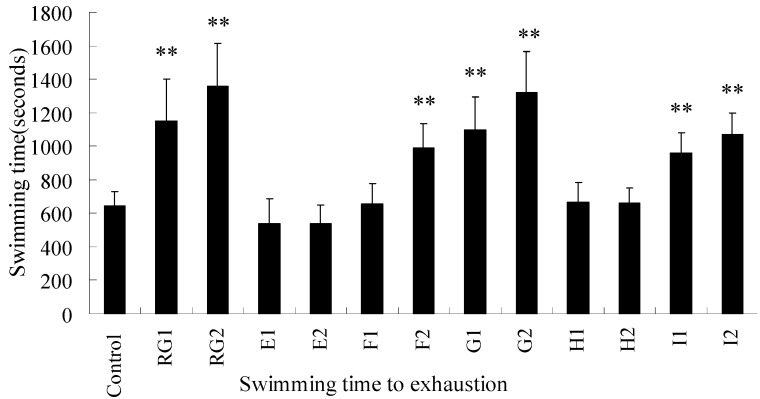
The effects of n-butanol fractions on the swimming time to exhaustion of weight-loaded mice. RG1: low dose of red ginseng, RG2: high dose of red ginseng, E1: low dose of water eluate, E2: high dose of water eluate, F1: low dose of 20% ethanol eluate, F2: high dose of 20% ethanol eluate, G1: low dose of 50% ethanol eluate, G2: high dose of 50% ethanol eluate, H1: low dose of 100% ethanol eluate, H2: high dose of 100% ethanol eluate, I1: low dose of 100% ethanol eluate, and I2: high dose of 100% ethanol eluate. The fractions were collected from D101 macroporous resin. Low dose denotes 10 mg/kg per mouse, and high dose denotes 20 mg/kg per mouse. I1 and I2 represent animals treated with nigaichigoside F1 at 2 mg/kg and 4 mg/kg, respectively.

We next examined the anti-fatigue effects of RPL and fractions on mice in a swimming-to-exhaustion test. In experiment I, the swimming time to exhaustion of mice receiving high-dose of the *n*-butanol fraction and the positive (red ginseng) group was significantly longer than that of the control group (Figure 2, *p* < 0.01). In experiment II, the swimming time of mice treated with high-dose of 20% ethanol, 50% ethanol fractions and nigaichigoside F_1_ was significantly improved (Figure 3, *p* < 0.01). Thus, RPL and its fractions are effective in increasing the swimming time to exhaustion, indicating the anti-fatigue activity of RPL compounds.

** indicates statistically significant difference as compared to the control group (*p* < 0.01).

** indicates statistically significant difference as compared to the control group (*p* < 0.01).

### 2.4. The Effects of RPL on SUN, TG, LDH, Ammonia, LA and HG

The concentrations of serum SUN, TG, LDH, ammonia and LA and hepatic glycogen (HG) serve as indicators of accumulated fatigue. As shown in Table 3 and Table 4, the SUN levels were significantly decreased in the red ginseng group (*p* < 0.01), the high dose ethyl acetate fraction group (*p* < 0.05) in the mouse experiment I, the low dose *n*-butanol fraction group (*p* < 0.01), the high dose *n*-butanol fraction group (*p* < 0.01), the high dose water residue group (*p* < 0.05), the low dose 50% ethanol eluate group (*p* < 0.05), the high dose 50% ethanol eluate group (*p* < 0.01), nigaichigoside F_1_ 4 mg/kg group and nigaichigoside F_1_ 2 mg/kg group (*p* < 0.05) in experiment II. The concentration of serum triglyceride (TG) in red ginseng, high-dose ethyl acetate, and low dose *n*-butanol fraction groups were lower than that in the control group (*p* < 0.05), with the high-dose *n*-butanol group being more highly significant (*p* < 0.01). In experiment II, the TG levels were also lower in red ginseng, the high-dose 20%, 50% ethanol fractions and nigaichigoside F_1_ groups as compared to control group (*p* < 0.05 or *p* < 0.01). LDH change was not significantly different in all groups in the experiment I. However, in the experiment II, among the groups of the high-dose 20% ethanol fraction, and both doses of 50% ethanol fractions and nigaichigoside F_1_, the LDH level was significantly higher in comparison with the untreated group (*p* < 0.05). The serum ammonia levels in the high dose n-butanol fraction group were significant lower than those in the control group (*p* < 0.05) in experiment I. 

**Table 3:** The effects of the fractions of RPL and red ginseng on SUN, TG, LDH and ammonia.

Groups	SUN (mmol/L)	TG (mmol/L)	LDH (IU/L)	Ammonia (umol/L)
Control	10.33 ± 0.63	2.880 ± 0.1052	585.1 ± 21.12	93.1 ± 7.29
Red Ginseng	9.29 ± 0.71 **	2.765 ± 0.1246 *	590.7 ± 17.22	91.9 ± 6.71
A1	9.95 ± 0.77	2.875 ± 0.0664	575.2 ± 23.64	94.9 ± 8.48
A2	9.74 ± 0.76	2.858 ± 0.1055	580.8 ± 21.80	98.3 ± 8.51
B1	9.81 ± 0.78	2.931 ± 0.1064	594.6 ± 28.41	87.2 ± 7.21
B2	9.04 ± 0.66 *	2.784 ± 0.0763 *	587.7 ± 28.23	90.5 ± 6.38
C1	9.41 ± 0.76 **	2.769 ± 0.0568 *	597.2 ± 21.50	88.1 ± 6.67
C2	8.84 ± 0.70 **	2.745 ± 0.0573 **	603.3 ± 27.45	85.6 ± 8.14 *
D1	9.72 ± 0.68	2.967 ± 0.0934	567.6 ± 23.28	94.7 ± 9.03
D2	9.55 ± 0.69 *	2.909 ± 0.1184	578.4 ± 24.91	95.2 ± 6.56

A1: low dose of petroleum ether fraction; A2: high dose of petroleum ether fraction; B1: low dose of ethyl acetate fraction; B2: high dose of ethyl acetate fraction; C1: low dose of *n*-butanol fraction; C2: high dose of n-butanol fraction; D1: low dose of water reside; D2: high dose of water reside; Low dose denotes 20 mg/kg; high dose denotes 40 mg/kg for each mouse; * *p* < 0.05 and ** p < 0.01 *vs*. control group.

In experiment II, among mice receiving high-dose 20% ethanol fraction, and both doses of the 50% ethanol fractions and nigaichigoside F_1_, their LDH levels were significantly higher in comparison with the untreated group (*p* < 0.05, *p* < 0.01). LDH was increased by *n*-butanol and nigaichigoside F_1_ fractions. LDH is known to catalyze lactic acid into pyruvate, thereby decreasing the accumulation of lactic acid in muscle to attenuate fatigue [10,14], although a previous report cited that liposoluble fraction from *Acanthopanax senticosus* decreased LDH [15]. The serum ammonia levels in mice treated with high-dose 20% ethanol fraction, and both doses of the 50% ethanol fractions and nigaichigoside F_1_ showed a significant decrease in experiment II, while in experiment I only high dose *n*-butanol fraction treated group showed a decrease in the serum ammonia levels. In experiment II, we also determined the levels of serum LA and HG, As shown in Table 4, the LA level was significantly decreased in the groups that were treated with high dose of red ginseng, 50% ethanol eluate and nigaichigoside F_1_ (*p* < 0.05 or *p* < 0.01), while the concentration of HG was higher (*p* < 0.05 or *p* < 0.01). These results confirm that RPL and its fractions possess anti-fatigue properties.

**Table 4:** The effects of the *n*-butanol and nigaichigoside F_1_ fractions on SUN, TG, LDH, ammonia, LA and TG.

Groups	BUN (mmol/L)	TG (mmol/L)	LDH (IU/L)	Ammonia (umol/L)	LA (mg /dL)	HG (mg/g)
Normal	6.48 ± 0.45	1.959 ± 0.069	383.9 ± 23.27	52.2 ± 5.09	44.1 ± 4.6	47.90 ± 5.83
Control	9.66 ± 0.68	2.162 ± 0.083	894.5 ± 35.59	134.1 ± 7.26	65.9 ± 6.1	19.44 ± 1.96
RG1	9.06 ± 0.86	2.077 ± 0.090 *	919.9 ± 60.71	128.9 ± 10.21	61.3 ± 5.6	39.26 ± 3.82 **
RG2	8.91 ± 0.71 *	2.034 ± 0.077 **	922.4 ± 49.74	127.4 ± 9.62	59.3 ± 6.4 *	43.17 ± 5.38 **
E1	9.38 ± 0.70	2.148 ± 0.077	927.5 ± 50.40	129.7 ± 9.32	62.2 ± 6.9	19.76 ± 1.93
E2	9.66 ± 0.84	2.134 ± 0.053	921.1 ± 43.57	127.7 ± 12.56	61.9 ± 8.7	20.20 ± 1.72
F1	9.41 ± 0.63	2.083 ± 0.123	908.3 ± 38.90	126.2 ± 10.50	64.6 ± 8.8	20.60 ± 2.47
F2	9.21 ± 0.75	2.052 ± 0.092 *	932.5 ± 42.57 *	121.1 ± 15.02 *	63.8 ± 8.3	19.30 ± 2.06
G1	8.90 ± 0.71 *	2.050 ± 0.096 *	938.2 ± 47.62 *	115.7 ± 12.94 **	57.8 ± 7.1 *	40.59 ± 4.19 **
G2	8.58 ± 0.69 **	1.992 ± 0.074 **	970.5 ± 81.40 *	104.1 ± 11.62 **	54.5 ± 6.3 **	43.04 ± 4.59 **
H1	9.30 ± 0.82	2.165 ± 0.104	903.2 ± 33.03	125.7 ± 14.64	63.4 ± 8.1	21.58 ± 3.34
H2	9.14 ± 0.98	2.178 ± 0.125	916.2 ± 30.97	135.1 ± 11.28	62.2 ± 7.1	21.89 ± 3.60
I1	9.09 ± 0.83	2.170 ± 0.103	937.0 ± 44.94 *	113.3 ± 11.54 **	60.9 ± 4.3 *	38.67 ± 3.24 **
I2	8.79 ± 0.76 *	2.067 ± 0.107 *	951.9 ± 58.00 *	109.0 ± 15.19 **	58.7 ± 5.7 *	41.45 ± 3.81 **

Normal: non-swimming control group; Control: swimming control group; RG1: low dose of red ginseng; RG2: high dose of red ginseng; E1: low dose of water eluate; E2: high dose of water eluate; F1: low dose of 20% ethanol eluate; F2: high dose of 20% ethanol eluate; G1: low dose of 50% ethanol eluate; G2: high dose of 50% ethanol eluate; H1: low dose of 100% ethanol eluate; H2: high dose of 100% ethanol eluate; I1 and I2 mean that animals were treated with nigaichigoside F_1_ 2 mg/kg and 4 mg/kg; * *p* < 0.05 and ** *p* < 0.01 *vs*. control group.

### 2.5. The Effects of RPL on IL-6 and TNF-α

Fatigue is associated with increased immune activation and the production of inflammatory cytokines [16,17]. Therefore, we analyzed two inflammatory cytokines in experiment II. As shown in Table 5, the serum level of IL-6 was significantly decreased in the groups that were treated with high dose of red ginseng, 50% ethanol eluate and nigaichigoside F_1_ (*p* < 0.05). The concentration of TNF-α was also lower in the groups receiving red ginseng, 50% ethanol fractions and nigaichigoside F_1_ groups as compared to control group (*p* *<* 0.05 or *p* < 0.01). These results confirm that RPL and its fractions possess anti-fatigue properties through suppression of pro-inflammatory responses.

**Table 5:** The effects of the *n*-butanol and nigaichigoside F_1_ fractions on IL-6and TNF-α.

Groups	IL-6 (pg/mL)	TNF-α (pg/mL)
Normal	224.19 ± 19.51 **	870.08 ± 53.65 **
Control	348.04 ± 25.39	978.43 ± 52.14
RG1	328.97 ± 17.46	926.13 ± 34.58 *
RG2	320.11 ± 19.94 *	905.50 ± 44.7 **
E1	334.61 ± 26.80	941.72 ± 47.44
E2	340.64 ± 18.67	945.96 ± 73.91
F1	328.69 ± 30.19	956.67 ± 53.94
F2	339.75 ± 26.34	945.39 ± 75.32
G1	317.34 ± 29.03 *	912.73 ± 35.94 **
G2	310.84 ± 32.74 *	907.71 ± 39.16 **
H1	337.22 ± 34.54	937.43 ± 45.74
H2	343.11 ± 33.55	947.29 ± 49.97
I1	325.76 ± 19.94 *	931.77 ± 38.32 *
I2	324.21 ± 17.17 *	923.91 ± 44.69 *

Normal: non-swimming control group; Control: swimming control group; RG1: low dose of red ginseng; RG2: high dose of red ginseng; E1: low dose of water eluate; E2: high dose of water eluate; F1: low dose of 20% ethanol eluate; F2: high dose of 20% ethanol eluate; G1: low dose of 50% ethanol eluate; G2: high dose of 50% ethanol eluate; H1: low dose of 100% ethanol eluate; H2: high dose of 100% ethanol eluate; I1 and I2 mean that animals were treated with nigaichigoside F_1_ 2 mg/kg and 4 mg/kg; * *p* < 0.05 and ** *p* < 0.01 *vs*. control group.

## 3. Experimental

### 3.1. Chemicals, Reagents and Instruments

The following reagents were used in this study: EtOH (AR), petroleum ether (AR), ethyl acetate (AR), and *n*-butanol (AR) (Chongqing Chuandong Chemical Co. Ltd., Chongqing, China). D101 macroporous resin was purchased from Chemical Plant of Nankai University (Tianjin, China), and prepared according to the manufaturers instructions. Briefly, the resin was immersed in 95% ethanol for 24 h, washed by distilled water, and then washed by 5% HCl, 5% NaOH and distilled water thoroughly [18] before use. HPLC analysis of calibration solutions and those of extracts and fractions of *Rubus parvifolius* L. (RPL) was performed on a Waters modular chromatograph (Waters Corporation, Milford, MA, USA). This system was controlled by Millennium 32 software. The system consisted of a Waters-2690 binary pump and a photo diode array detector (PDA) model 996. HPLC separations were performed on a YMC-Pack ODS-AM column (4.6 × 250 mm, 5 μm) (YMC Co. Ltd., Tokyo, Japan) and a YMC-Pack ODS-AM column (10 × 250 mm, 10 μm). The samples were eluted with MeOH and a solution containing ultrapure H_2_O (Milli-Q, Millipore, Billerica, MA, USA) and formic acid (FA, 1%). ^1^H and ^13^C-NMR spectra were obtained on a Bruker Avance-500 spectrometer (Bruker, Karlsruhe, BW, Germany). 

### 3.2. Plant Materials

The whole plants of RPL were obtained from Chongqing Huiyuan Pharma. Co., Ltd. (Chongqing, China) Red ginseng pieces were purchased from Chongqing Tongjunge Pharmacy Chainstore (Chongqing, China). All plants were authenticated at the Chongqing Institute of Chinese Materia Medica.

### 3.3. Extraction, Isolation of RPL Components

**Figure 4:**
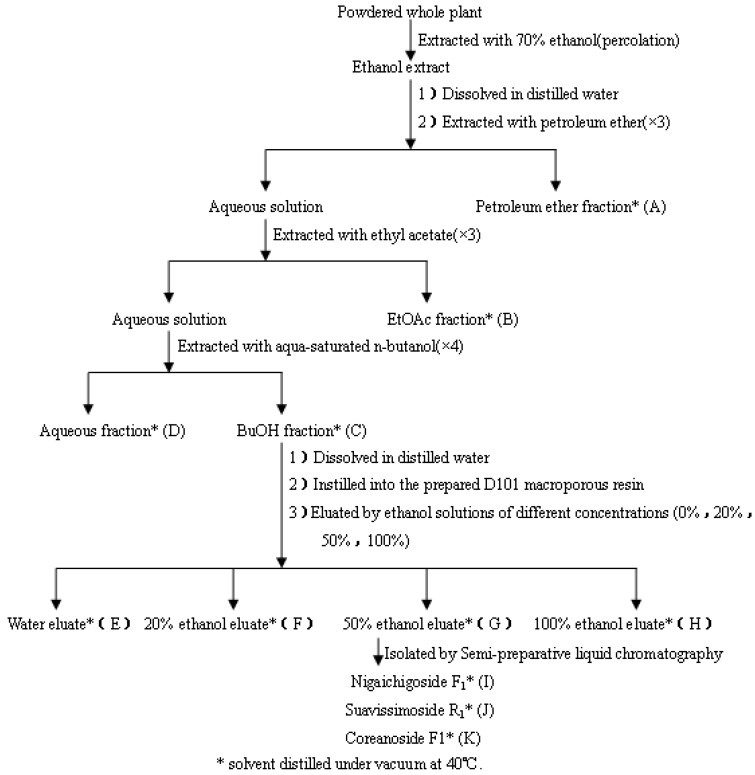
Schematic description of the procedures for extraction and fractionation of the whole plant of *Rubus parvifolius* L.

Powdered whole plant of RPL (20 kg) was extracted and fractionated as illustrated the flow chart in Figure 4. Powdered whole plant of RPL (20 kg) was soaked in 70% ethanol aqueous solution (40 L) for 24 h, and then extracted using 10 volumes of 70% ethanol aqueous solution by percolation, the percolation rate was 300 mL/(kg·min) extraction. The solvent was evaporated under vacuum to yield the crude extract (1,752 g). This extract was then suspended in water and partitioned successively with petroleum ether (3 × 5 L), ethyl acetate (3 × 5 L) and water-saturated *n*-butanol (4 × 5 L). Each fraction was evaporated to dryness under reduced pressure to afford the petroleum ether (A, 74 g), ethyl acetate (B, 451 g), *n*-butanol (C, 446 g) and aqueous (D, 603 g) residues. The extracts were concentrated under vacuum at 40 °C, lyophilized to obtain powders, and then used as test samples.

The TLC profile of the *n*-butanol fraction mainly showed the presence of saponins, thus the *n*-butanol fraction was purified and enriched through D101 macroporous resin chromatography. The residue of the *n*-butanol fraction was suspended in distilled water, then the solution was slowly added onto the D101 macroporous resin, at a rate of 2,000 mL/h, to ensure that the sample was completely adhered to the resin. After that the resin was washed with distilled water until there was no sugar in the elute, and then eluted with ethanol solutions of different concentrations (20%, 50% and 100%) successively at 500 mL/h.

Each desorbed solution was evaporated *in vacuo* to afford the water eluate (E, 216 g), 20% ethanol eluate (F, 72 g), 50% ethanol eluate (G, 88 g) and 100% ethanol eluate (H, 44 g) residues. The 50% ethanol eluate residue (8 g) was purified by semi-prep-RP-HPLC with a YMC-Pack ODS-AM column (1% formic acid-methanol, 55:45; flow rate: 2 mL/min; injection volume: 50 µL; sample concentration: 100 mg/mL in methol; UV conditions: 208 nm) allowing the isolation of 25 mg of compound I, 16 mg of compound J and 11 mg of compound K (Figure 1A–D).

*2α,3β,19α,23-Tetrahydroxyurs-12-ene-28-O-β-D-glucopyranosyl ester (nigaichigoside F_1_*, **I**) (Figure 1C) was obtained as a white powder. ^1^H-NMR (500 MHz, C_5_D_5_N, δ ppm) 1.61 (3H, s, CH_3_-27), 1.40 (3H, s, CH_3_-24), 1.28 (3H, s, CH_3_-29), 1.22 (3H, s, CH_3_-26), 1.05 (3H, s, CH_3_-25), 1.02 (3H, d, *J* = 6.6 Hz, CH_3_-30), 4.53–3.97 (6H, glucose), 2.88 (1H, s, H-18), 4.25 (1H, m, H-2), 4.75 (1H, d, *J* = 9.0 Hz, H-3), 5.51 (1H, t-like, H-12), 6.18 (1H, d, *J* = 7.5 Hz, H-1′); ^13^C-NMR (125 MHz, C_5_D_5_N, δ ppm) 47.2 (C-1), 67.9 (C-2), 78.4 (C-3), 43.2 (C-4), 47.3 (C-5), 17.8 (C-6), 32.6 (C-7), 40.4 (C-8), 47.1 (C-9), 36.9 (C-10), 24.1 (C-11), 127.8 (C-12), 138.7 (C-13), 41.5 (C-14), 28.7 (C-15), 26.4 (C-16), 47.9 (C-17), 53.8 (C-18), 72.2 (C-19), 41.5 (C-20), 26.1 (C-21), 37.1 (C-22), 65.9 (C-23), 14.0 (C-24), 17.2 (C-25), 17.1 (C-26), 24.3 (C-27), 176.5 (C-28), 26.6 (C-29), 16.3 (C-30), 95.3 (C-1′), 73.3 (C-2′), 77.9 (C-3′), 70.7 (C-4′), 78.1 (C-5′), 61.8 (C-6′).

*2α,3β,19α -Trihydroxyurs-12-ene-23-carboxyl-28-O-β-D-glucopyranosyl ester (suavissimoside R_1_*, **J**) was obtained as a white amorphous powder. ^1^H-NMR (500 MHz, C_5_D_5_N, δ ppm) 1.73 (3H, s, CH_3_-27), 1.62 (3H, s, CH_3_-24), 1.36 (3H, s, CH_3_-29), 1.20 (3H, s, CH_3_-26), 1.16 (3H, s, CH_3_-25), 1.09 (3H, d, *J* = 6.6 Hz, CH_3_-30), 2.87 (1H, s, H-18), 4.32 (1H, m, H-2), 4.62 (1H, d, *J* = 9.4 Hz, H-3), 5.27 (1H, t-like, H-12), 6.27 (1H, d, *J* = 8.1 Hz, H-1′); ^13^C-NMR (125 MHz, C_5_D_5_N, δ ppm) 48.2 (C-1), 68.3 (C-2), 80.7 (C-3), 54.2 (C-4), 52.1 (C-5), 21.5 (C-6), 33.3 (C-7), 40.2 (C-8), 47.7 (C-9), 38.3 (C-10), 24.2 (C-11), 127.8 (C-12), 138.8 (C-13), 41.8 (C-14), 28.8 (C-15), 26.1 (C-16), 47.8 (C-17), 53.9 (C-18), 72.3 (C-19), 41.6 (C-20), 26.0 (C-21), 37.2 (C-22), 179.4 (C-23), 13.3 (C-24), 17.1 (C-25), 16.8 (C-26), 24.4 (C-27), 176.3 (C-28), 26.5 (C-29), 16.1 (C-30), 95.5 (C-1′), 73.5 (C-2′), 78.2 (C-3′), 70.9 (C-4′), 78.5 (C-5′), 61.9 (C-6′).

*Coreanoside F_1_* (**K**) was obtained as a white powder. ^1^H-NMR (500 MHz, C_5_D_5_N, δ ppm) 1.05 (3H, d, *J* = 6.7 Hz, CH_3_-30′), 1.09 (3H, d, *J* = 6.1 Hz, CH_3_-30), 1.76–1.10 (9 singlets: CH_3_-29, CH_3_-29′, CH_3_-27, CH_3_-27′, CH_3_-26, CH_3_-26′, CH_3_-25, CH_3_-25′, CH_3_-24), 2.86 (1H, s, H-18′), 2.87 (1H, s, H-18), 3.99 (1H, d, *J* = 10.1 Hz, H-23′b), 4.13 (1H, ddd, *J* = 4.1,9.4,10.3 Hz, H-2), 4.24 (1H, d, *J* = 9.4 Hz, H-3), 4.68 (1H, d, *J* = 10.2 Hz, H-23′a), 5.08 (1H, br, *J* = 10.4 Hz, H-2′), 5.52 (1H, t-like, H-12′), 5.53 (1H, t-like, H-12), 6.14 (1H, d, *J* = 7.5 Hz, GlcII-H-1′), 6.15 (1H, d, *J* = 7.3 Hz, GlcI-H-1), 6.51 (1H, br, H-3′).

For pharmacological studies, fractions A, B, C, D, E, F, G, H, I were dissolved in 1% aqueous Tween-80. The doses used are expressed as mg of the dried extract per kg body weight.

### 3.4. Animals

Five-week-old male Kunming mice (20 ± 2 g) were kept at 25 ± 1 °C and 55% ± 10% relative humidity with 12 h of light (artificial illumination: 08:00–20:00). Animal study was conducted in accordance with the guidelines for the Ethical Treatment of Laboratory Animals (License No. SYXK (Yu) 2007-0002). All procedures of mouse care and handling were in accordance with accepted standard operating procedures of the Third Military Medical University. Animals study was approved by the Animal Care and Utilization Committee of the Third Military Medical University.

### 3.5. Experimental Design

#### 3.5.1. Weight-Loaded Swimming Experiment I

Mice were trained to be accustomed to swimming once (for 10 min) in the first week. To reduce the variations in the swimming capacity, mice capable of swimming with 30% longer or shorter time than the average with similar swimming time and body weights were excluded. Mice were randomly grouped into ten groups of each containing ten animals. Mice in the treatment groups with four RPL fractions (petroleum ether, ethyl acetate, *n*-butanol, and aqueous fractions from the aqueous ethanol extract were administered with two doses, 40 mg/kg and 20 mg/kg respectively [4]. The positive experimental groups were treated with red ginseng (40 mg/kg).

The RPL extracts were administered orally daily for consecutive 30 days. In order to make the animals accustomed to swimming, training (no loaded on the tails) was carried out once every 3 days. Thirty minutes after the last oral administration, the swimming-to-exhaustion test was carried out as previously described [19,20,21]. Briefly, mice were dropped individually into an acrylic plastic pool (90 cm × 50 cm × 50 cm) filled with fresh water at 25 ± 1 °C at 40 cm deep so that the mice could not touch the bottom with their tails to support themselves. A lead block (4% of body weight) was attached to the tail root of each animal. The swimming time to exhaustion was used as the index of the forced swimming capacity. The mice were assessed as exhausted when they failed to rise to the surface of water for breath within a 10 s period.

#### 3.5.2. Analysis of Blood Biochemical Parameters

Blood samples were collected without anticoagulant after measurement of the swimming time and cooled for about 3.5 h at 4 °C. The serum was prepared by centrifugation at a speed of 1,000 × g, 4 °C for 20 min and the levels of serum urea nitrogen (SUN), serum triglyceride (TG), serum lactate dehydrogenase (LDH) and serum ammonia were quantified using commercial kits supplied by Beckman Coulter, Inc. (Brea, CA, USA).

#### 3.5.3. Weight-Loaded Swimming Experiment II

Based on the similar swimming time and body weights, mice were randomly divided into fourteen groups, including one non-swimming control group, one swimming control group, two positive control groups and ten treatment groups. Each group contained ten mice. The red ginseng treatment positive control groups were administered with 40 mg/kg and 20 mg/kg respectively. The treatment groups with the different eluates collected from D101 macroporous resin were gavaged with 20 mg/kg and 10 mg/kg eluates respectively [22]. Nigaichigoside F_1_ was applied at 2 or 4 mg/kg. The doses were selected based on the results of preliminary experiments. The measurement of the weight-loaded swimming test and biochemical analysis of serum were similar to experiment I with the exception that extracts of RPL were administered orally daily for a consecutive 15 days and a lead block (3% of body weight) was loaded on the tail root of the mice. After that, the levels of serum urea nitrogen (SUN), serum triglyceride (TG), serum lactate dehydrogenase (LDH), lactic acid (LA), serum ammonia and hepatic glycogen (HG) were determined by the automatic biochemical analyser with commercial kits. Two inflammatory cytokines, interleukin-6 (IL-6) and tumor necrosis factor (TNF-α) in the serum were also examined.

### 3.6. Statistical Analysis

All values are expressed as the denotes ± S.E.M. All statistical analyses were performed using SPSS software (SPSS for Windows 16.0, SPSS Inc., Chicago, IL, USA). Statistical difference was determined by one-way ANOVA, followed by LSD-t test for multigroup comparisons. *p <* 0.05 was considered statistically significant.

## 4. Conclusions

To assess the effect of RPL on physical fatigue and identify the effective component(s) in RPL, we carried out bioactivity-guided fractionation and selected a mouse weight-loaded swimming test for examination of the extent of physical fatigue. The study confirms the anti-fatigue effect of RPL and the active components of RPL to be its total saponins. Nigaichigoside F_1_ is at least partly responsible for the pharmacological effect of RPL. The possible mechanisms of alleviation of physical fatigue by RPL may involve delays in the accumulation of SUN and LA, a decrease in the level of TG by increasing fat utilization, increases in the levels of HG and LDH to reduce the accumulation of lactic acid, a reduction of the blood ammonia concentration in the muscle, and suppressions of increments of immune activation and inflammatory cytokines. Further studies are needed for isolation of possible novel compounds from RPL with anti-fatigue activity.
